# Returning Home after Decontamination? Applying the Protective Action Decision Model to a Nuclear Accident Scenario

**DOI:** 10.3390/ijerph19127481

**Published:** 2022-06-18

**Authors:** Joel Rasmussen, Petter B. Wikström

**Affiliations:** 1 Crisis Communication Centre, School of Humanities, Education and Social Sciences, Örebro University, 70182 Örebro, Sweden; 2 Department of Economics, School of Business, Economics and Law, University of Gothenburg, 40530 Gothenburg, Sweden; petter.bjornson@gmail.com

**Keywords:** nuclear accidents, decontamination, risk perceptions, return migration, local populations, PADM

## Abstract

Studies of the aftermath of nuclear power plant accidents show that affected citizens assess higher risks and adopt more risk-avoidant behaviors than authorities expect. This results in differences between the planned recovery and actual outcomes. Based on this knowledge, this study examined the factors that affect citizens’ preference to continue living in a decontaminated area. Testing the key aspects of the protective action decision model (PADM), this study analyzed Swedish survey data (*N* = 2291) regarding such an accident scenario. Several aspects of the PADM, from the layperson’s view of threats and protective actions, to stakeholders and situational factors, were strongly supported. The most influential variables affecting settlement choices are perceptions of radiation risk, perceptions of decontamination effectiveness, government information, living with certain restrictions, and attachment to an area because of one’s work. A novel contribution of this study is that it ranked the significance of such effects on behavioral intentions in an emergency scenario. Regarding the policy recommendations, this study concluded that a recovery program must facilitate most aspects of people’s lives and provide trustworthy information on decontamination efficiency. As some people will avoid potential health risks and leave a decontaminated area, planning to implement one solution for everyone would likely not be optimal.

## 1. Introduction

In recent decades, research on disaster risk governance and management has emphasized the importance of involving various stakeholders in the decision-making processes to increase the probability that the measures will be accepted and effective [1,2,3,4]. Similarly, risk communication scholars argue that an awareness and understanding of public concerns must be at the core of effective risk and emergency communication [5]. In the case of preparedness for nuclear accidents, initiatives have been established in nine European countries to involve stakeholders in facilitating recovery planning [6]. However, accidents in the nuclear energy system leading to nuclear fallout remain, perhaps, the most challenging disaster scenario, with consequences that cannot be insured against [7,8]. Stakeholder-participation initiatives rarely involve those laypeople who may be affected, whose perceptions of risk have been shown to differ from those of the experts [9,10], and upon whose behaviors the authorities depend in emergency management and recovery [11].

In a nuclear accident scenario, a major uncertainty is how citizens will act [11]. From an expert perspective, the average person tends to overreact to the risk of radiation exposure [12]. For instance, if authorities instruct citizens to shelter in their homes after an incident, many will still voluntarily evacuate [11,13]. If successful decontamination has taken place, that is, efforts to remove radioactive material and thereby sufficiently reduce the radiation dose rate, and the authorities are urging evacuees to return, a large proportion prefer to stay in their new locations and avoid their old hometowns, because of perceived radiation risk and emotional distress [14,15]. If risks are categorized on a scale all the way from negligible to intolerable [16], sizable groups of citizens see exposure to radiation as intolerable, even at low doses [10]. With the Fukushima Daiichi accident, for example, even when all of the financial support for the evacuees had ceased and economic conditions were such that they compelled citizens to return to their homes, 47% of the affected population did not return [17]. Being female, young, having children [18,19], and having a higher socioeconomic status [20] correlated with moving permanently from areas near the Fukushima Daiichi accident. Simultaneously, the authorities’ high ambitions for reconstruction were not fully realized. With many citizens not returning, the cost of decontamination per returning citizen rises steeply, which was shown after the Fukushima Daiichi accident. The decontamination cost per returnee has been estimated at 3.36 million USD [18]. In addition, demographic decline and imbalance negatively affect a region’s economy [21], which fuels further regression.

These are relevant concerns for those countries operating nuclear power plants. With 443 nuclear power reactors in 30 countries, and 50 new facilities under construction [22], all countries arguably need to understand what public behavior is to be expected if the release of radioactive materials occurs from a reactor and have contingency plans that are not naïve about the public’s reactions [23]. Given that restoration projects depend on citizens retaining their homes and rebuilding a community, the purpose of this present study is to examine and rank the factors that influence citizens to want to live in an area that has undergone remediation after a nuclear accident. Such knowledge can inform risk management and risk communication initiatives in ways that incorporate those aspects that citizens highly value, resulting in recovery programs that are more accepted and achieve greater success.

This study analyzed survey data (*N* = 2291) from Sweden, a country that operates nuclear power plants that supply 39% of the country’s electricity [24]. The analysis drew on one of the most well-established theories dealing with citizens’ emergency behaviors, the Protective Action Decision Model (PADM) [12,23], which, to our knowledge, had yet to be applied to the Northern European region.

## 2. Literature Review 

### 2.1. The Protective Action Decision Model (PADM)

The PADM is one of a few influential theories that explain how decisions are made in risky situations [25]. Other theories, such as the theory of planned behavior [26,27] and the theory of protection motivation [28], focus more on individual risks and personal health contexts. Designed for and applied to collective risks and mass emergencies, the PADM is particularly useful for understanding behaviors in a nuclear accident scenario, which is the context in which it was first applied [13,25]. However, this model has also been used in studies on other types of collective risks, such as wildfires [29], chemical release from the petrochemical industry [30], city smog [31], flood hazards [32], and the ‘not-in-my-backyard’ attitude to nuclear power plants [33].

The PADM combines a wide range of factors that influence household decisions if a threat occurs. Overall, this theory assumes that people’s access to and assessment of cues in and information from their living environments, social contacts, media, information channels, and risk messages play a role in how they value and manage risk. People’s characteristics, including demographic data, also play a role. Central to the model is the assessment of exposure, and thus susceptibility, to risk. This stage includes what it is about a risk that people pay attention to and comprehend. Based on this, it is assumed that citizens form perceptions about the severity of a risk, about the feasibility and effectiveness of measures, and about the actors who are involved in the management of a risk. More than one measure may seem reasonable. If a nuclear accident occurs that threatens a community, the stakeholders not only include the authorities and the media, but also the independent experts, the company that operates a nuclear plant, and all of the affected households [25]. Taken together, these considerations are assumed to shape protective action decisions. However, preferred decisions are also conditioned in everyday life. The model includes situational factors that facilitate or hinder the realization of desired decisions.

The scope of the model also means that most of the studies focus on parts of it or specialize in certain areas, such as risk communication [30] or evacuation modeling [34,35]. Our paper notes Lindell and Perry’s [25] remark that “it is still not entirely clear what motivates people to take protective action”. Although a set of factors is highly relevant, we know less about whether risk perception is a strong determinant vis-à-vis protective action perceptions or other considerations. More accurate knowledge could inform risk communication efforts, so the issues that most likely ‘move’ an issue from audiences’ perspectives are also the ones that are emphasized in government communication. We limited this study’s focus to examining the extent to which a preferred place of residence after decontamination is affected by perceptions of (a) threats, (b) protective action, (c) stakeholders, and (d) the situational factors that facilitate or impede a preferred action.

### 2.2. Research on Citizens’ Responses and This Study’s Research Hypotheses

Extant research demonstrates that certain demographic factors affect the decisions to move or stay after nuclear accidents that lead to radioactive contamination. Those who, to a greater extent than others, tend to leave areas affected by radioactive material and do not return are women, parents with at-home children, and people with higher socioeconomic status [18,19,36,37]. Women and parents expressed greater concern about the negative health effects of ionizing radiation, which justified risk-avoidant behavior, such as settling somewhere not affected by a radioactive release [14,15,36,38]. Beyond the demographic factors, such as various contributing aspects that can be actualized using the PADM, the state of knowledge is more uncertain.

According to lay opinion, radioactive material disseminated in connection with nuclear power accidents is particularly dangerous [39,40]. They rate it at the top among other so-called dread risks [10,41], and as a possible hazard for decades to come [18]. Citizens perceive ionizing radiation as particularly unpleasant and dangerous, because they associate it with disasters in which many have been negatively affected and the outcomes have sometimes been cancer and fatalities. They also perceive radioactive substances as difficult to control, even for the expert authorities [9]. It was also shown after the Fukushima accident in Japan that perceptions of high risk and concern about ionizing radiation predict a greater tendency to move from a decontaminated area [13,17,18,36]. There is no reason to believe that a similar association would not be found in the Swedish scenario.

**Hypothesis** **1.** *The perception of more serious threat characteristics is associated with less propensity to return to live in a decontaminated area.*

A study about citizens from the Fukushima prefecture showed that awareness of the remediation area having been declared safe from radiation benefited relocation [17], which was expected. However, the same study tempers the expectations of return rates after decontamination because it also shows that the cancellation of evacuation orders after remediation had a much lesser effect than the termination of allowances for housing and living expenses for evacuees [17], indicating that household finances play a more significant role than decontamination. Another study showed that decontamination had a statistically significant effect on the return level, but that the measure itself caused less than 8% of all of the evacuees to return [18]. These two studies thus suggest that decontamination has a statistically significant but still rather weak effect. Citizens’ uncertainty regarding the health effects of low-dose radiation achieved with decontamination can be sufficient motivation to not return [14,36]. Regarding food safety after the Fukushima accident, it was shown that those who believe that the current safety standards in Japan are sufficient are less risk-averse when it comes to protective action decisions concerning food [42]. Like food control, decontamination is carried out based on safety standards.

**Hypothesis** **2.** *Belief in the effectiveness of decontamination is associated with a greater propensity to return to live in a decontaminated area.*

In different ways, the studies have focused on the importance of stakeholder perceptions. A study by Tateno and Yokohama that focused on the Fukushima residents showed that mistrust of responsible government institutions mediates worry over radiation [43]. Another study found that a sense of injustice and being treated unfairly—thus lower trust in responsible government agencies—motivates citizens not to follow the recommendations to return [38]. Zhang et al. concluded that distrust of government information is associated with permanent migration [14]. An analysis of qualitative interview data further adds that governments and authorities can be seen as remote bureaucracies trying to decide in a crisis in which they bear no health risk [44].

**Hypothesis** **3.** *Trust in information from government agencies is associated with a greater propensity to return to live in a decontaminated area.*

Citizens’ assessments of the importance of social relationships influence settlement-related decisions [36]. In a study on citizens’ reasons not to return from a temporary housing community, Orita concluded: “The most commonly articulated reason for staying in temporary housing was the sense of community that they had built” [36]. However, if someone has family members living in a decontaminated area, the chance of returning increases [17]. In other types of crises, social ties have also proved to be important for household decision-making [45]. Stakeholder perceptions thus include citizens’ assessments of the importance of their peers’ positions on the issue of whether to return.

**Hypothesis** **4.** *People who attach importance to others’ assessment of the settlement choice are likelier to return to live in a decontaminated area.*

Finally, decisions on where to settle involve several factors that may impede or facilitate a preferred settlement choice. Studies have found that individuals’ finances are significant for their choice of residence after a nuclear accident. In areas that have not been as affected by radioactive contamination as others, such as coastal areas, a high socioeconomic status predicts a higher return rate [37]. However, in more heavily contaminated areas, a high socioeconomic status predicts a lower return rate [18,20]. Thus, if finances were not a concern, more people would be likely to stay away from contaminated neighborhoods. A lack of financial resources has also been shown to prevent people from moving from radiation-affected areas during the year following an accident [20]. This importance of finances was also demonstrated when canceled evacuation orders did not significantly increase the rate of relocation, but the termination of financial compensation and housing subsidies for evacuees did [17]. Thus, the household economy is one of several situational factors that affect settlement decisions. In particular, a lack of financial resources prevents a number of citizens from making their preferred settlement choices. Owning property and depending on a job in an affected area predict a greater likelihood of returning [17,44], but the same could apply if better means of subsistence exist elsewhere. Zhang et al. demonstrated that people are motivated to move permanently if better job-market opportunities are identified elsewhere [14]. Results from an interview study similarly demonstrated that a better job market and educational opportunities in cities not affected by a nuclear accident motivated young adults to migrate [38].

**Hypothesis** **5.** *If people feel attached to their hometown through work, schools, investments, and so on, they are likelier to return to live there after decontamination.*

Just as access to work is important for settlement decisions, so are schools and other community services [17].

## 3. Materials and Methods

To examine and rank the factors that influence citizens to prefer living in an area that has undergone remediation after a nuclear accident, we analyzed survey data collected during the period 22 February 2019 to 28 March 2019 about laypersons’ assessments of a nuclear accident scenario. The survey was sent out by e-mail by the Laboratory of Opinion Research (LORE) to 3800 adult Swedish citizens (a maximum of three reminders were sent), of whom 2291 participated. The sample was stratified by gender, age, and education. The sample errors consisted of some instances of missing data. Between 2% and 6% of the units lacked a response to some of the questions studied and were excluded. Therefore, the response count (*N*) varied slightly from one question to another. The significance level was set at *p <* 0.05. We measured the statistical significance of the effect of value changes in independent variables on the preference to return and keep living in the decontaminated area with a Z-test, employing the standard normal distribution. The null hypothesis equals zero change from the independent variable’s base level. Likelihood Ratio (LR) χ^2^ tests were also performed to test the overall significance of the models.

Before the respondents answered the survey questions, we presented a hypothetical scenario in which a nuclear accident had occurred in Sweden and the respondents’ neighborhoods had been affected by nuclear fallout and undergone remediation. The scenario description is available in Appendix A. We dropped all of the answers from respondents who spent less than five seconds reading the instructions (46 respondents) since it was below the minimum time frame to grasp the scenario.

### 3.1. Response Variables

To study the settlement preferences in the context of a nuclear power plant (NPP) accident and recovery scenario, we examined the factors that affect the following dependent variable: the likelihood of returning to live in one’s home after it has been declared safe (Table 1). Since the PADM is ultimately about decision-making when at risk, the decision that is at the center of our study is the choice to stay in a decontaminated area (or, alternatively, move). This particular preference was measured using the response alternatives “not at all likely”, “not very likely”, “somewhat likely”, and “very likely”. All of the factors possibly influencing this decision (except the controls) were also measured on a four- or five-point Likert scale, very similar to how the PADM has been studied for several decades [13]. For some variables, the dependent variable was flipped so as to instead describe the likelihood of not staying. This concerns the variables for threat perception and for one of the variables measuring situational facilitators and impediments (expected value-loss of property). This was completed in order to facilitate the comparison between the sets of questions concerning estimated magnitudes. It is noted in the tables in the results section when this was implemented.

### 3.2. Variables of Interest

We investigated the impact of *threat perceptions* on the protective action decision-making using the following variables: ‘To what extent do you feel worried about the idea of living in a residential area with surrounding green areas that cannot be used for hunting, mushroom and berry picking, and play due to elevated radiation levels?’; ‘To what extent would you be concerned about radioactive substances in your home, even though measurements show that the levels are harmless to your health?’; and ‘How threatening would it be if your residential area were affected by a nuclear fallout?’ The response alternatives for all of the variables are shown in the results section.

We investigated the impact of *protective action perceptions* using the following variables: ‘To what extent do you believe authorities can restore housing to a safe level through decontamination?’; ‘Would it be reasonable for authorities not to decontaminate houses that show radiation levels below the limit values?’; ‘To what extent do you trust authorities to communicate correct information about a cleanup?’

When exploring *stakeholder perceptions,* we chose in this study to focus on citizen perceptions of the government agencies mainly responsible for the cleanup, as well as significant others and acquaintances [25]. The responses on three survey items were then analyzed, consisting of the extent to which respondents would take up other people’s views on the settlement decision after the cleanup including: (1) relatives; (2) government agencies; and (3) friends and acquaintances. Perceptions of other stakeholders, such as news media, were considered more difficult to measure in a valid way, since our scenario did not include respondents being exposed to case-specific news reporting.

We investigated the impact of *situational facilitators and impediments* using the following variables: ‘How attached to your hometown is your household through investments?’; ‘How attached to your hometown is your household because of work?’; ‘How attached to your hometown is your household through spare-time interests?’; ‘What do you think would happen in the short term with the value of properties in the residential area after the nuclear accident?’

### 3.3. Control Variables

The control variables were the same for all of the regressions and include gender, age, education, individual income, and whether a respondent had one or more children in the household (see Table 2).

### 3.4. Empirical Strategy

The analysis consisted of performing an ordered logistic regression (ordered logit) since the variables were categorical and ordered. The ordered logit model builds on the concept of latent regression $y^{*}=X^{\prime }\beta +\varepsilon$, where y* is a continuous variable that expresses the respondents’ true opinions but that is unobserved since the respondents answer according to a set of discrete alternatives. We, however, observed the discrete values of y*, and we called these values the categorical variable *y*, constructed from the respondents’ answers. That is, the assumed continuous variable y* manifests itself in different response categories of the observed dependent variable y. Hence, we assumed that if y* was within a certain interval, then the corresponding response category was chosen by the respondent. The ordered logit model estimates the likelihood of how the levels of β are associated with the changes in the dependent variable. The error terms are assumed to be distributed according to a logistic distribution, normalized to a mean, and variances of zero and one [46].

Our results are expressed as adjusted odds ratios (AORs), which estimate an associated change in the dependent variable by a change in the independent variables compared to the base level, while holding all of the other independent variables constant. The AOR is hence the multiple that describes how many times higher the odds are of a dependent variable “jumping” upwards from an increase in an independent variable. The underlying assumption of the model is that all of the aforementioned changes in the odds, between higher and lower categories, are the same, and this is called the proportional odds assumption.

Because all of the independent variables in this paper are ordinal, they were split into several binary variables, where the lowest level of the variables was used as a base level or reference category. The estimated associations hence describe the “move to that specific category from the base level”. The results, therefore, describe the associated change in the odds by moving from, for example, “a very low extent” to “a very high extent” of agreement. Hence, all of the categories will have effect sizes relative to the base level. That the AORs give the relative effect size means that they only provide a change in the odds and hence say nothing about the absolute value of the odds. Of course, a large change in the small initial odds will not yield a large absolute effect, even if it is a large relative effect.

## 4. Results

The results were divided into a series of tables separately describing the effects of threat perceptions, protective action perceptions, stakeholder perceptions, and situational facilitators and impediments, concluding with a ranking of the effects.

### 4.1. Threat Perceptions

Table 3 demonstrates that the perception of a greater threat is associated with an increased likelihood of not staying in a decontaminated neighborhood. The table presents the effects that responses above the base value of the independent variables have on preference levels to not stay in the decontaminated area (i.e., a flipped dependent variable). The columns containing the estimated AORs and *p*-values are accompanied to the left by a description of the different levels of the corresponding independent variable.

The variable measuring the uneasiness of living in an area with surroundings that cannot be used freely proved to have a strong association with the preferences not to stay and statistically significant results for all of the values except one. Assessing the situation to be uncomfortable to a somewhat large extent compared to a minuscule extent increases the odds of being more likely not to stay by a factor of 16.22, which is surpassed by the change in the odds of going from ‘the very small extent’ response to ‘the very large extent’ response, which multiplies the odds by a factor of 81.87. The results regarding concern over radiation when an area has been declared safe exhibit an even stronger association with a preference not to stay. Being concerned to a somewhat large extent increases the odds of being more likely not to stay by a factor of 30.72, and moving from the base level to a very large extent produces the very large factor of almost 152.

The column on the far right shows the association between the assessed degree of a threat if radioactive fallout affects one’s residential area and the likelihood of not staying. The lowest two values show non-statistically significant results. Yet, the highest values are statistically significant and associated with an increased probability of not staying, at a factor of 2.43.

### 4.2. Protective Action Perceptions

Table 4 demonstrates the effect of protective action perceptions on settlement preferences, with the dependent variable now set in the original direction, and hence measuring the likelihood of staying. The results for belief in the decontamination effectiveness show highly significant, positive, and large effects across the response categories. Going from a very small extent to a somewhat small extent multiplies the odds of a stronger preference for staying by approximately 3.69 times. Going from a very small extent to a very large extent multiplies the odds of being more likely to stay by a large factor of 74.98 times.

As seen in the column on the far right, believing it to be reasonable that authorities do not decontaminate houses below limit values increases the odds of staying. Regarding trust that government agencies provide the correct information, the results are generally highly significant throughout, and in a positive direction. For example, going from a very small extent to a very large extent regarding trust that governmental agencies give correct information multiplies the odds of a higher likelihood of staying by 7.76 times.

### 4.3. Stakeholders’ Perceptions

Table 5 shows the variables we chose to gauge the stakeholder perception facet of the PADM. The effects are highly significant and positive (i.e., considering others’ perspectives on settlement decisions is associated with a larger likelihood of staying).

Listening to the authorities has by far the largest effect. Moving from the base level to very large extent multiplies the odds of a higher likelihood of staying by more than 16 times. This is in line with the strong result for trust in government communication shown in Section 4.2. Interestingly, the results were positive throughout. This means that considering others’ viewpoints is associated with an increase in the likelihood of staying, even though the question is ambiguous as to these other actors’ recommendations. It is also interesting that listening to relatives’ and friends’ views to a large extent implies a slightly reduced association with the preference to return and live in the decontaminated area.

### 4.4. Situational Facilitators and Impediments

As shown in Table 6, we found generally significant and positive results for the variables that proxy situational facilitators or impediments with similar magnitudes. People are more likely to stay if their households are attached to an area through investments, their occupations, or their spare-time interests. However, the respondents who believed that property values would decrease equivalent to a large loss showed a strong likelihood of not staying, at a multiple of 7.83, with statistically significant results for the highest response category. The three leftmost variables are analyzed with the dependent variable, and the rightmost with the flipped dependent variable.

### 4.5. Rankings of Magnitudes

Table 7 shows the approximate order of magnitude (in terms of a statistically significant effect, where the insignificant effects were treated as zeros). The magnitudes were collected from the analyses using the controls.

## 5. Discussion

Nuclear accidents followed by remediation involve difficult judgments for decision-makers and affected populations. On the one hand, with current policies stating that decontamination and relocation should take place [47], authorities have a defined path to follow. If decontamination can lower radiation doses below health-hazard limits, their goal is for an area to become vital again. On the other hand, some risks are feared by the public, such as the emission of radioactive material caused by nuclear accidents [8]. Studies have reported high levels of worry among citizens and a propensity among many to leave the areas that authorities have planned to recover [14,17]. This makes decontamination particularly expensive per returning person [18], while at the same time those who do not return have little opportunity for financial support.

The novel contribution of our study is that it ranked the association between citizens’ assessments of a post-accident remediation scenario and their settlement intentions. Since the variables relate to key facets of the PADM, this study also provides evidence for how relevant the theoretical model is for a NPP accident and recovery scenario. Just as Lindell and Perry [25] predicted with the PADM, the perceptions of threat, protective action, and stakeholders proved to have a significant impact on one’s willingness to live in a decontaminated area. Thus, our results largely confirm Hypotheses 1–4. The perception of more serious threat characteristics proved to have the strongest effect of all, the top AOR being 151.94, and is associated with a lower propensity to keep living in a decontaminated area. Belief in the effectiveness of decontamination and trust in the information from governmental agencies are associated with a greater propensity to keep one’s home. Belief in decontamination efficiency can increase one’s willingness to live in a remedied area by as much as a factor of 74.98. Thus, decontamination efficiency is an issue with a great potential to change attitudes toward decontaminated areas. Uptake of others’ views on settlement decisions is also associated to a degree with a greater likelihood of keeping one’s home in the decontaminated area. Listening to others indicates some agreeableness, which could be expected to be associated with following the authorities’ advice to relocate. Confirming our fifth hypothesis, the situational facilitators (e.g., being attached to the area through work and investments) affect one’s propensity to stay in a decontaminated area. However, concerns about declining property values are a situational impediment to staying.

The effect of protective action perceptions that our study demonstrated marks a difference compared to the studies focusing on Fukushima [18,37], which found that decontamination has a statistically significant but rather weak effect on citizens’ return rates. It is difficult to discern the cause of this difference. One reason may be the Japanese government’s increase of the permitted radiation dose to 20 mSv/y, which caused criticism [48] and may have led to a decrease in public confidence that decontamination efforts resulted in environments that were safe enough to live in again. Another reason may be that our study is scenario-based and that the respondents underestimated the restrictions that apply even in a successfully decontaminated area, even though they were informed about them when taking the survey.

The fact that not everything can be cleaned up and that restrictions must apply in certain places significantly reduces one’s willingness to live in a remediated area. There is, thus, resistance to living a life with limitations and to facing health risks if these limits are not respected. Gosh and Boyd [49] stated that the recovery plans presume that citizens will take individual responsibility to monitor risks and behaviors in this way, potentially without a time limit. Our results show that these circumstances, which require citizens to continuously practice caution, constitute an incentive to start over elsewhere, where life can continue without perpetuated and individualized risk management. It is not that life with restrictions is necessarily dreaded, but relative to living without them, they seem to decrease the quality of life and motivate migration. Two consequences that are relevant to risk and to emergency planners may be extracted from these results. First, it seems advisable to focus on the effectiveness of the remediation in communicating with an affected audience. This issue can move public attitudes toward settlement choices. Second, communication does not seem to be enough to greatly increase one’s willingness to live in a remediated area, but more comprehensive redevelopment that allows a freer life outside and in green areas would be needed.

This study shows results highlighting that restoration needs to be a holistic effort. Beyond decontamination and radiation levels, issues related to household investments, work opportunities, and one’s social network help to facilitate (or impede) preferred settlement choices. These factors can have effects that play in favor of either staying or moving. If people experience staying as a better economic solution, and if community services are sufficient and they have a social life there, they will be motivated to stay. However, the opposite can apply if an area is perceived as stuck in decline, caused by contamination and stigma. Our results indicate that without compensation for material losses, those who own houses may be forced to stay for financial reasons. As others have shown [18,20], it is also likely that those with a sufficiently high socioeconomic status will use their resources to live where they prefer and migrate to a greater extent than others. Overall, in this Northern European case study, we saw great similarities with the results of the research from Fukushima, in that economics and social ties play major roles in where people settle after decontamination [14,17,38,44].

Finally, it must be acknowledged that this study does not present the most robust data, since they are based on a hypothetical scenario. We still hope that we have shown that the data and analysis are valuable because we also need knowledge about areas where no major nuclear accident has occurred. In addition, previously found associations between intentions and behaviors [50] and the similarities with the results from Japan (e.g., [14]) strengthen the results of this current study. Another possible weakness of the application of the PADM [25] is that different studies apply the model slightly differently as they focus on various risks, and they rarely test the entire model since it is so wide in scope. This can be seen as weakness in terms of validity. Nevertheless, we believe that it may be more important for the validity of the research that studies are adapted to new contexts of risk exposure than that a study design is repeated with great accuracy. If the assumptions of the PADM are confirmed even with somewhat differently designed testing, its validity is arguably strengthened, as was the case in this study. Furthermore, even if individual studies do not succeed in testing the whole model, parts of it can be examined, which is still valuable. Larger projects may take a more holistic approach in the future.

Further research is needed to build knowledge about how realistic and humane recovery might be organized. The possibilities for compensation appear to be a major question if people can be able at all to make their preferred settlement choices. Research into this is necessary. With such issues integrated into emergency planning, a process will likely gain significantly higher acceptance and goal achievement.

## 6. Conclusions

In examining the factors that affect citizens’ preferences to keep living in a decontaminated area after an RN accident, this study largely confirms the assumptions expressed in the PADM model. It also adds novel knowledge by specifying the effect sizes of the various factors that influence settlement decisions. Several facets of the PADM (e.g., perceptions of threat, protective action, and stakeholders) proved to be significant for the settlement choices in a NPP accident and decontamination scenario. The results show that the most influential variable in a choice of residence is the attitude to radiation risk, followed by belief in the decontamination’s effectiveness. The concerns over radiation risk after decontamination are a very potent, negative factor. The perceptions of stakeholders have positive effects of varying magnitudes, with uptake of the authorities’ view of the settlement decision having the strongest positive association with the settlement choice. Finally, we found that factors beyond the specifics of decontamination and radiation levels play a major role. Investments in a recovery area and access to jobs, schools, and social networks strongly influence the settlement decisions.

Based on the results, three lessons for emergency planning are suggested. First, the current standards for redevelopment may need to be raised to increase public acceptance, with strict limit values and extensive redevelopment around the residential areas to curb worry and allow a freer life. Second, the effectiveness of decontamination appears to be a key aspect about which to communicate for the emergency management, since it is an issue that has the potential to shape attitudes toward a recovery area. Finally, given public concern about low-dose radiation, material losses, and the limitations on leisure activities that some will experience, it would likely be beneficial to consider compensating victims so that households can choose their futures more freely, thereby also reducing the risk that authorities and NPP operators will lose valuable trust.

## Figures and Tables

**Table 1:** Dependent variable summary statistics.

Variable	Levels	Numerical Value	*N*	Freq.	Mean	Median	Std	Min	Max
The likelihood of staying after a home is declared safe			2185		2.38	2	0.87	1	4
	Not at all likely	1		15.79%					
	Not very likely	2		40.73%					
	Somewhat likely	3		33.50%					
	Very likely	4		9.98%					

**Table 2:** Summary statistics of demographic variables.

Variable	Levels	*N*	Freq.	Mean	Median	SD	Min	Max
Sex, binary	1 female, 0 male	2245	48.20% (female)	0.48	n/a	n/a	0	1
Age categories, categorical	6 levels. <30, 30–39, 40–49, 50–59, 60–69 and ≥70	2142	n/a	3.81	4 (50–59 yrs.)	1.61	<30	≥70
Education categories, categorical	9 levels. See the Appendix B for details.	2143	n/a	6.36	7 (university < 3 yrs.)	2.04	No completed educ.	PhD
Income categories, personal, categorical	12 levels. See the Appendix B for details.	2051	n/a	7.27	8 (30,000–36,999 SEK)	2.59	<4000 SEK	≥65,000 SEK
Children ≥ 1, binary	Binary: 1 = one or more children in the household; 0 = no children in the household	2148	27.75% (≥1 child)	0.28	n/a	n/a	0	1

**Table 3:** Effect of threat perceptions on the likelihood of not staying.

Flipped Dependent Variable: The Likelihood of Not Staying after Decontamination				
Levels of Independent Variables	Uneasiness of Living Where Nature Cannot Be Used Freely	Concern over Radioactive Substances in the Home	Levels of Independent Variable	Threatening If Radioactive Fallout Affects Your Neighborhood
Very small extent	(Base level)	(Base level)	Not at all threatening	(Base level)
Somewhat small extent	1.60	3.97 ***	Not very threatening	1.42
	0.375	0.000		0.188
Neither small nor large extent	3.66 **	8.75 ***	Somewhat threatening	0.99
	0.009	0.000		0.972
Somewhat large extent	16.22 ***	30.72 ***	Very threatening	2.43 ***
	0.000	0.000		0.000
Very large extent	81.87 ***	151.94 ***		
	0.000	0.000		
Controls	Yes	Yes		Yes
Observations	1947	1948		1942
LR χ2	649.04 (0.000)	860.28 (0.000)		156.37 (0.000)

*p*-values in parentheses, ** *p* < 0.01, *** *p* < 0.001.

**Table 4:** The effect of protective action perceptions on the likelihood of staying.

Dependent Variable: Likelihood of Staying after Decontamination				
Levels of Independent Variables	A Belief that Governmental Agencies Can Restore Residences via Decontamination	Trust that Governmental Agencies Can Communicate Correct Information	Levels of Independent Variable	Reasonable that Governments Do Not Clean Houses below Limited Values
Very small extent	(Base level)	(Base level)	Very unreasonable	(Base level)
Somewhat small extent	3.69 ***	1.48	Somewhat unreasonable	2.23 ***
	0.000	0.220		0.000
Neither small nor large extent	6.85 ***	3.04 ***	Somewhat reasonable	3.36 ***
	0.000	0.000		0.000
Somewhat large extent	15.56 ***	4.66 ***	Very reasonable	4.95 ***
	0.000	0.000		0.000
Very large extent	74.98 ***	7.76 ***		
	0.000	0.000		
Controls	Yes	Yes		Yes
Observations	1943	1944		1947
LR χ2	540.76 (0.000)	207.70 (0.000)		177.16 (0.000)

*p*-values in parentheses, *** *p* < 0.001.

**Table 5:** The effect of stakeholders’ perceptions on the likelihood of staying.

Dependent Variable: The Likelihood of Staying after One’s Residence Has Been Declared Safe			
Levels of Independent Variables	Uptake of Relatives’ Viewpoints about Settlement Decision	Uptake of Governmental Agencies’ Viewpoints about Settlement Decision	Uptake of Friends’ Viewpoints about Settlement Decision
Very small extent	(Base level)	(Base level)	(Base level)
Somewhat small extent	1.97 **	3.11 ***	2.84 ***
	0.002	0.000	0.000
Neither small nor large extent	3.23 ***	8.12 ***	3.83 ***
	0.000	0.000	0.000
Somewhat large extent	2.82 ***	13.48 ***	3.13 ***
	0.000	0.000	0.000
Very large extent	2.54 ***	16.30 ***	2.53 ***
	0.000	0.000	0.000
Controls	YES	YES	YES
Observations	1944	1937	1937
LR χ2	130.67 (0.000)	386.35 (0.000)	146.11 (0.000)

*p*-values in parentheses, ** *p* < 0.01, *** *p* < 0.001.

**Table 6:** The impact of situational facilitators and impediments on the likelihood of staying or not.

Dependent Variable: Likelihood of Staying after Decontamination				Flipped Dependent Variable: Likelihood of not Staying after Decontamination	
Levels of Independent Variables	Attachment to an Area through Investments	Attachment to an Area through Work	Attachment to an Area through Spare-Time Interests	Levels of Independent Variable	Expected Value-Loss of Property
Not at all decisive	(Base level)	(Base level)	(Base level)	Largely unchanged	(Base level)
Not very decisive	3.52 ***	3.28 ***	3.24 ***	Reasonable loss	0.97
	0.000	0.000	0.000		0.973
Somewhat decisive	5.56 ***	6.72 ***	5.45 ***	Significant Loss	2.99
	0.000	0.000	0.000		0.142
Very decisive	8.67 ***	12.23 ***	6.29 ***	Large loss	7.83 **
	0.000	0.000	0.000		0.006
Controls	YES	YES	YES	Controls	Yes
Observations	1720	1753	1851	Observations	1947
LR χ2	231.05 (0.000)	338.79 (0.000)	311.97 (0.000)		221.84 (0.000)

*p*-values in parentheses, ** *p* < 0.01, *** *p* < 0.001.

**Table 7:** Rankings of magnitudes.

Variable	Mean Odds Ratio	Facet of PADM	Mean Change
Concern over radioactive substances in the home	48.85	Threat perception (flipped dep. var.)	4785%
Uneasiness if nature cannot be used freely	25.44	Threat perception (flipped dep. var.)	2469%
Belief in decontamination effectiveness	25.27	Protective action perception	2427%
Uptake of authorities’ view on settlement decision	10.25	Stakeholder perception	925%
Attachment to an area through work	7.41	Situational facilitator	641%
Attachment to the area through investments	5.92	Situational facilitator	492%
Attachment to an area via leisure activities	4.99	Situational facilitator	399%
Believes govt. agencies’ information is correct	3.87	Protective action perception	312%
Reasonable if homes below hazard limits are not cleaned	3.51	Protective action perception	251%
Uptake of friends’ view on settlement decision	3.08	Situational facilitator	208%
Uptake of relatives’ views on settlement decision	2.64	Stakeholders’ perceptions	164%
Expected value-loss of property	2.61	Situational impediment (flipped dep. var.)	228%
Threatening if radio-nuclide fallout affects your neighborhood	0.81	Threat perception (flipped dep. var.)	48%

## Data Availability

Third party data. Restrictions apply on the availability of these data. Data were obtained from The Laboratory of Opinion Research (LORE) and are available from the lead author with the permission of LORE.

## Appendix A. Translated Transcript of a Scenario

A nuclear accident has occurred in Sweden, and your residential area is contaminated with radioactive substances. You have been evacuated to temporary housing while authorities are cleaning up parts of your residential area. Remediation has included removal of contaminated land close to your house, cleaning, and removing radioactive material from roof and facade surfaces, and, if necessary, indoor surfaces. The disposal of adjacent land is likely to damage vegetation, such as flowerbeds. Remediation measures can take up to one year to complete.

After the cleanup, measurements of houses and gardens show that the levels of radioactive substances are so low that they are considered harmless. However, there are areas around your residential area that show levels of radiation so high that you are not allowed to live there, and in some cases, they require special permits for access. Authorities have advised parents not to allow their children to play freely in surrounding natural areas. They also advise against hunting and berry and mushroom picking. Some industries (especially hunting, fishing, and agriculture) may find that selling certain products is prohibited or difficult. The following questions are designed to discover your sentiments about living in such a residential area.

## Appendix B. Detailed Descriptive Statistics of the Control Variables

**Variable**	**Explanation**	**Levels**	***N***	**Freq.**	**Mean**	**Median**	**SD**	**Min**	**Max**
Female	Gender of respondent. Binary.		2245		0.48	0	0.50	0	1
		0. Male		51.80%					
		1. Female		48.20%					
Age groups	Age groups, categorical.		2142		3.81	4	1.61	<30	≥70
		1. <30		9.20%					
		2. 30–39		16.15%					
		3. 40–49		18.21%					
		4. 50–59		17.37%					
		5. 60–69		19.51%					
		6. ≥70		19.56%					
Edu.	Education levels, categorical.		2143		6.36	7	2.04	1	9
		1. No completed elementary school	3	0.14%					
		2. Elementary school	71	3.31%					
		3. High school or eq. < 3 years	191	8.91%					
		4. High school or eq. ≥ 3 years	279	13.02%					
		5. Post-secondary, non-university, <3 years	235	10.97%					
		6. Post-secondary, non-university, ≥3 years	62	2.89%					
		7. University < 3 years	278	12.97%					
		8. University ≥ 3 years	911	42.51%					
		9. PhD	113	5.27%					
Incp.	Monthly personal income (before tax) groups. Categorical.		2051		7.27	8	2.59	<4000	>65,000
		1. <4000 SEK	46	2.24%					
		2. 4000–8999 SEK	38	1.85%					
		3. 9000–12,999 SEK	148	7.22%					
		4. 13,000–15,999 SEK	101	4.92%					
		5. 16,000–18,999 SEK	122	5.95%					
		6. 19,000–25,999 SEK	270	13.16%					
		7. 26,000–29,999 SEK	230	11.21%					
		8. 30,000–36,999 SEK	410	19.99%					
		9. 37,000–44999 SEK	323	15.75%					
		10. 45,000–54,999 SEK	178	8.68%					
		11. 55,000–64,999 SEK	78	3.80%					
		12. >65,000 SEK	107	5.22%					
Children ≥ 1	One or more children in the household. Binary.		2148		0.28	0	0.45	0	1
		0: No children	72.25%						
		1: One or more	27.75%						
